# Cell-free fat extract restores hair loss: a novel therapeutic strategy for androgenetic alopecia

**DOI:** 10.1186/s13287-023-03398-1

**Published:** 2023-08-23

**Authors:** Yizuo Cai, Zhuoxuan Jia, Yichen Zhang, Bijun Kang, Chingyu Chen, Wei Liu, Wei Li, Wenjie Zhang

**Affiliations:** 1 Department of Plastic and Reconstructive Surgery, Shanghai 9th People’s Hospital, Shanghai Jiao Tong University School of Medicine, Shanghai Key Laboratory of Tissue Engineering, National Tissue Engineering Center of China, 639 ZhiZaoJu Road, Shanghai, 200011 China; 2 Department of Biological and Environmental Engineering, Cornell University, Ithaca, USA

**Keywords:** Androgenetic alopecia, Fat extract, Cell-free therapy

## Abstract

**Background:**

Androgenetic alopecia (AGA) is one of the most common hair loss diseases worldwide. However, current treatments including medicine, surgery, and stem cells are limited for various reasons. Cell-free fat extract (CEFFE), contains various cell factors, may have potential abilities in treating AGA. This study aims to evaluate the safety, effectiveness and the underlying mechanism of CEFFE in treating AGA.

**Methods:**

Sex hormone evaluation, immunogenicity assay and genotoxicity assay were conducted for CEFFE. In vivo study, male C57BL/6 mice were injected subcutaneously with dihydrotestosterone (DHT) and were treated with different concentration of CEFFE for 18 days (five groups and *n* = 12 in each group: Control, Model, CEFFE^Low^, CEFFE^Middle^, CEFFE^High^). Anagen entry rate and hair coverage percentage were analyzed through continuously taken gross photographs. The angiogenesis and proliferation of hair follicle cells were evaluated by hematoxylin–eosin, anti-CD31, and anti-Ki67 staining. In vitro study, dermal papilla cells (DPCs) were incubated with different concentrations of CEFFE, DHT, or CEFFE + DHT, followed by CCK-8 assay and flow cytometry to evaluate cell proliferation cycle and apoptosis. The intracellular DHT level were assessed by enzyme-linked immunosorbent assay. The expression of 5α-reductase type II, 3α-hydroxysteroid dehydrogenase and androgen receptor were assessed through reverse transcription-quantitative polymerase chain reaction (RT-qPCR) or/and western blot.

**Results:**

In CEFFE-treated mice, an increase in the anagen entry rate and hair coverage percentage was observed. The number of CD31-positive capillaries and Ki67-positive cells were increased, suggesting that CEFFE promoted the proliferation of DPCs, modulated the cell cycle arrest, inhibited apoptosis caused by DHT, reduced the intracellular concentration of DHT in DPCs, and downregulated the expression of AR.

**Conclusions:**

CEFFE is a novel and effective treatment option for AGA through producing an increased hair follicle density and hair growth rate. The proposed mechanisms are through the DHT/AR pathway regulation and regional angiogenesis ability.

**Supplementary Information:**

The online version contains supplementary material available at 10.1186/s13287-023-03398-1.

## Background

Androgenetic alopecia (AGA) is a common multifactorial dermatologic condition characterized by progressive hair loss across frontotemporal and vertex scalp regions [1, 2]. The highest prevalence was found in Caucasians, and the morbidity rate gradually increases with aging [3, 4]. Although the specific mechanisms underlying AGA remains incompletely understood, the excess testosterone metabolite dihydrotestosterone (DHT) derivate from testosterone and high expression of androgen receptors (AR) are vital mediators in AGA development [5–7]. DHT binds to AR in dermal papilla cells (DPCs) regulating downstream target gene transcription, resulting in hair follicle degeneration [1, 6]. Specifically, the rapid growth phase (anagen) was shortened, and the no-growth phase (telogen) was prolonged. Over time, the terminal hair was converted into thinner and shorter villus hair [8]. Thus, altering the DHT or AR expression in DPCs is critical in androgen induced balding. Angiogenesis might also play a part in AGA, which the scalp's vascularity may be an essential environmental factor in the health of hair roots.

Currently in the market, orally taken Finasteride, and topically applied Minoxidil are used as the standard first-line treatments for AGA [9]. Finasteride, a selective steroidal inhibitor of 5-α-reductase could inhibit the conversion from testosterone to DHT, resulting in a decrease in serum and scalp DHT levels [10]. However, long-term usage of Finasteride is associated with rare complications like decreased libido, gynecomastia and psychologic impairments [11]. Minoxidil, on the other hand, induce vasodilation of peripheral vessels and improve microcirculation, leading to DPCs proliferation [12]. Nevertheless, topically applied Minoxidil is also absorbed into the systemic circulation and caused cardiovascular side effects [13]. Alternatively, hair follicle transplantation another way to treat AGA but is limited by the shortage of donor hair follicles, and the cost of treatment [14]. Therefore, more effective and economical approaches with fewer side effects are in urgent need to prevent AGA progression and stimulate hair regrowth.

Other than drug treatments, multipotent stem cells, such as adipose-derived stem cells (ADSCs), have been employed as attractive options for AGA therapy [15–17]. According to previous studies, stem cells mainly exert their therapeutic effects through a paracrine manner rather than differentiation into specific cell types [18, 19]. Those secreted factors, including hepatocyte growth factor (HGF), vascular endothelial growth factor (VEGF), and insulin-like growth factor (IGF), could promote angiogenesis, maintaining hair in anagen phase, and therefore activating hair growth [20–23]. However, the clinical application of such stem cell therapy is still limited by manufacturing issues and ethical concerns.

We propose using a novel economical therapeutic agent, cell-free fat extract (CEFFE), to treat AGA. CEFFE is the liquid fraction isolated that mechanically from liposuction wastes. According to previous studies, the composition of CEFFE is similar to stem cell paracrine factors, including VEGF, IGF, and HGF, which these factors promote neovascularization and have anti-inflammatory effects [24–28]. In addition, previous study has demonstrated CEFFE as a promising therapeutic strategy for treating inflammatory disease and ischemia disorders in the limb ischemia model, tissue expansion model, and diabetic wound healing model [28, 29]. The abundant sources, ease of acquisition, and low immunogenicity of CEFFE makes clinical application possible. Thus, we proposed that CEFFE has positive effects on regulating DHT/AR signaling and modifying the angiogenesis microenvironment of AGA patients. Both in vitro and in vivo experiment has been carried out, using DHT-induced AGA models, to evaluate the protective effects of CEFFE and investigate its underlying mechanism.

## Methods

### CEFFE preparation

The study was approved by The Ethics Committee of Shanghai Ninth People’s Hospital, Shanghai Jiaotong University School of Medicine, Shanghai, China (SH9H-2018-T22-1). After obtaining consent forms, adipose tissue was harvested from the abdomen or thigh of healthy adult females using liposuction from December 2020 to November 2021. In this study, CEFFE was derived from 5 females aged from 22 to 35. The preparation procedure of CEFFE followed previously established protocol [24]. Briefly, the harvested adipose tissue was flushed with physiological saline and centrifuged at 1200* g*/4 °C for 3 min 2 or 3 times to remove tissue debris, blood, and oil. Then, the emulsified fat was obtained by mechanical emulsification through two 10 mL syringes (KDL, China) connected by a three-way stopcock with an internal diameter of 2 mm (Terumo Corporation, Japan) 60 times. Immediately after, the emulsified fat was centrifuged at 1200* g*/4 °C for 5 min, and the liquid in the bottom was withdrawn. Finally, the extract supernatant was filtrated with a 0.22 μm filter (Corning, USA) as CEFFE and subsequently frozen at − 80 °C for future experiments [24]. The protein concentration in CEFFE was determined with a bicinchoninic acid assay kit (BCA, Thermo Fisher Scientific, USA).

### Safety evaluation of CEFFE

Considering that hormones (especially testosterone) may have an effect on AGA treatment, we collected CEFFE from 8 male patients, in which testosterone and estradiol were measured. Basic patient information was shown in Additional file 1: Table S1. For the immunogenicity and genotoxicity of CEFFE, we have done a series of tests based on ISO/IEC 17025. (1) In vitro cytotoxicity test. L929 cells were cultivated in blank control (10% FBS in α-MEM medium, Gibco, UAS), negative control (complete medium containing 10% polyethylene), positive control (complete medium containing 10% DMSO, Sigma, USA), experimental groups (complete medium containing 12.5%, 25%, 50%, 100% CEFFE, respectively) 24 h and cell viability were determined by MTT assay. Cell viability less than 70% was considered potentially cytotoxic. (2) Intracutaneous reactivity test. Three healthy adult male New Zealand White rabbits (China Institute of Food and Drug Verification, China) weighing > 2 kg were used for the test. 0.2 mL of CEFFE was intradermally injected at five points on one side of each rabbit's spine and 0.2 mL (0.9% NaCl; blank control) was intradermally injected in the same operation on the opposite side, with 1 cm between the injection points. 24, 48, 72 h after injection, erythema and edema at the injection sites were recorded. Each rabbit was euthanized with an intravenous injection of sodium pentobarbital (100 mg/kg, China Institute of Food and Drug Verification, China) after recording. The scoring method and reference standard can be referred to [25]. If the final score was ≤ 1, the sample was considered negative. (3) Systemic toxicity assay. A single dose of 50 mL/kg CEFFE was injected intraperitoneal in male Kunming mice (The weight at the time of injection was 17–23 g, and the difference between groups did not exceed ± 20% of the mean. China Institute of Food and Drug Verification, China) in the experimental group (*n* = 5), while 0.9% NaCl was applied in the control group (*n* = 5) according to the random number table. The animals were observed immediately following injection, 24, 48, and 72 h after application to evaluate the presence of toxic signs (weight loss, and/or death). All mice were euthanized with carbon dioxide inhalation, and the bilateral thoracotomy was used as second method to confirm the death. (4) The pyrogen test. 10 mL/kg CEFFE was injected to three healthy young adult male New Zealand White rabbits (China Institute of Food and Drug Verification, China) weighing > 1.7 kg. The baseline rectal temperature was recorded every 30 min for one hour before the injection. After one hour, rabbits were inoculated in the ear marginal vein. Temperatures were recorded every 30 min for three hours after the injection. Each rabbit was euthanized with an intravenous injection of sodium pentobarbital (100 mg/kg, China Institute of Food and Drug Verification, China) after recording. The test indicates the presence of a pyrogen when one of the three rabbits shows an increase in body temperature higher than 0.6 °C or when the sum of the maximum temperature rises in the three individual rabbits exceeds 1.3 °C. (5) Bacterial assay for gene Mutation-Ames test. Salmonella typhimurium histidine-requiring strains TA97, TA98, TA100, and TA102 were exposed to negative control, positive control and CEFFE respectively, the specific system was shown in Additional file 1: Table S5. The number of colonies in the positive control group should be more than 3 times that of the negative control group. If the number of colonies in the experimental group increases more than twice compared with the negative control group, it is a positive reaction. (6) Mouse lymphoma TK assay (MLA assay). Specific steps could be referred to [26], and the specific system was shown in Additional file 1: Table S6.

### Animals and experimental design

All animal experiments were approved by the Ethics Committee of Shanghai Jiao Tong University (SH9H-2021-A183-SB) carried out according to *ARRIVE* guidelines (https://www.nc3rs.org.uk/arrive-guidelines). Sixty male C57BL/6 mice (5–6 weeks old, 23.02 ± 2.0 g) were purchased from Charles River (Charles River Laboratories, China) and raised under standard conditions with constant temperature and humidity under 12-h phase light–dark cycle. Drinking water and food were freely available. During the experiment, all mice were kept on the same cage shelf in an animal room.

**Fig. 1:**
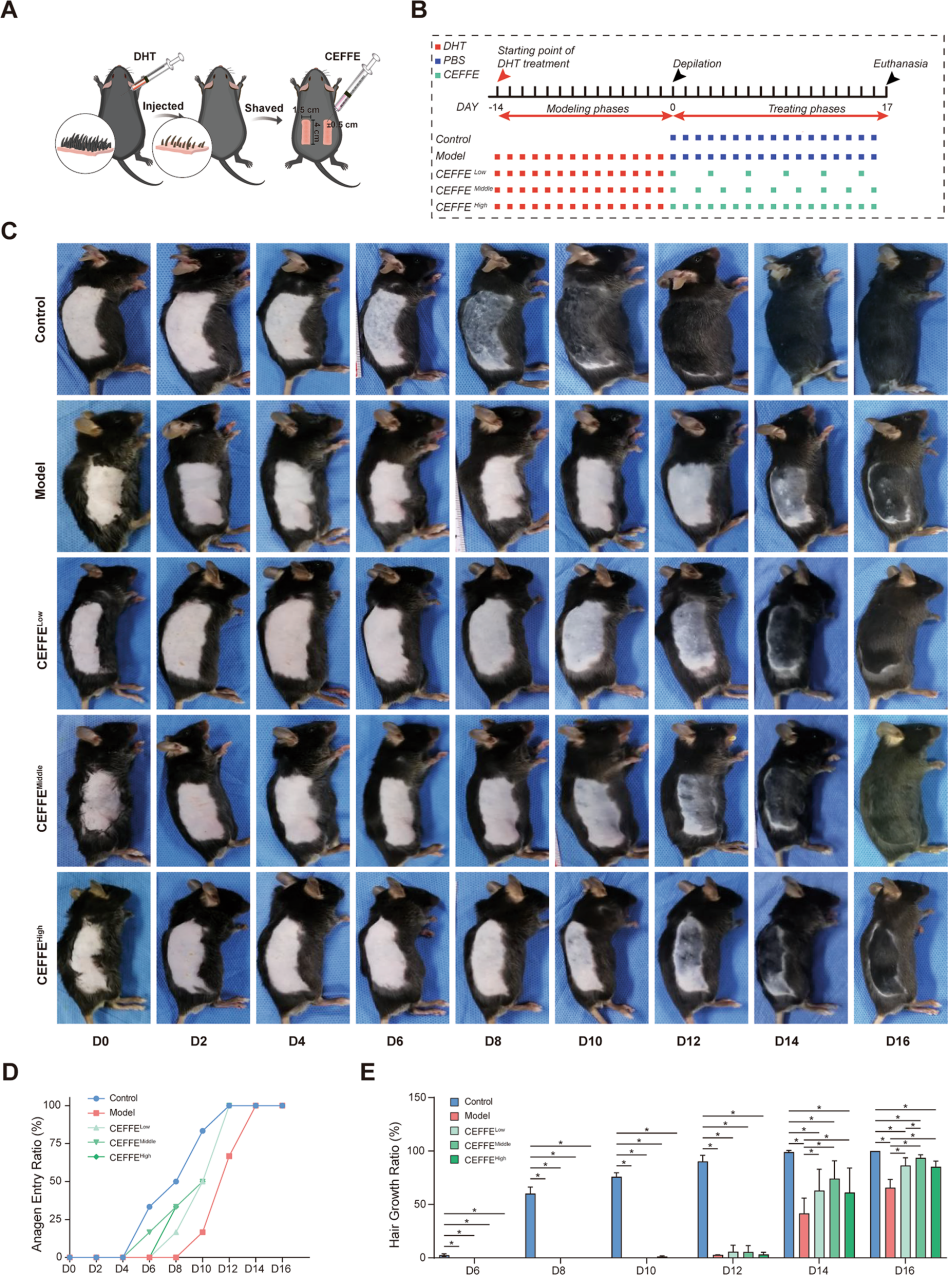
Therapeutic efficacy of cell-free fat extract (CEFFE) in dihydrotestosterone (DHT)-induced androgenic alopecia (AGA) C57BL/6 mouse models. **A** Schematic diagram of experimental design for CEFFE treatment in DHT-induced AGA C57BL/6 mouse models. **B** Flowchart of the dosing regimen and behavioral experiments. **C** Representative dorsal skin images of different groups from D0 to D16. **D** Anagen entry ratio (%) and corresponding timeline from D0 to D16. **E** Hair growth ratio (%) on the dorsal skin for each group from D6 to D16. Statistical Data represent the mean ± SD; *n* = 12; *P < 0.05

Group-specific methods and treatments were presented in Fig. 1A, B and summarized below. The sample size was calculated based on our pre-experimental results. Mice were divided into five groups randomly (*n* = 12 in each group) according to the random number table: Control, Model, CEFFE^Low^, CEFFE^Middle^, and CEFFE^High^. The experiment was divided into two consecutive phases: a modeling phase (D-14 to D-1) and a treatment phase (D0 to D16). During the modeling phase, subjects in all other groups except the control group were injected subcutaneously at the nape of the neck with DHT (2 mg/kg) every day [30, 31]. To synchronize the hair cycle [32, 33], both sides of all mice’s dorsal areas (4 × 1.5 cm^2^) were shaved using an electric clipper followed by depilatory cream to remove residual hair on D0. Entering the treatment phase, all subjects in the Control and Model group were injected subcutaneously with 100 μL PBS per day into the shaved areas. Mice in the CEFFE^Low^, CEFFE^Middle^, and CEFFE^High^ groups were treated with 100 μL CEFFE (3 μg/μL) at 3 days, 2 days, and 1 day interval respectively. All mice were euthanized with carbon dioxide inhalation, and the bilateral thoracotomy was used as second method to confirm the death on the 17th day, and the center region of previously shaved dorsal skin was acquired for further histology analysis.

### Macroscopic measurement for hair growth

To assess the hair growth process, photos of the hair-shaved areas were continuously obtained every two days from D0 to D16 using a Canon 70D digital camera (Canon Inc., Japan). The change of skin color from pink to grey indicated the transition of hair follicles from the telogen phase into the anagen phase [34]. Therefore, the date of first observing the skin turning grey was recorded, and the percentage of anagen entry rate (%)for each group was calculated as follows:

$${\text{Anagen}}\;{\text{entry}}\;{\text{rate}}\;\left( \% \right) = \frac{{{\text{Number}}\;{\text{of}}\;{\text{mice}}\;{\text{with}}\;{\text{grey}}\;{\text{skin}}}}{12} \times 100\% .$$

In addition, the hair coverage percentage of each group was calculated by Image J and represented as follows:

$${\text{Hair}}\;{\text{coverage}}\;{\text{percentage}}\;\left( \% \right) = \frac{{{\text{Sum}}\;{\text{of}}\;{\text{the}}\;{\text{area}}\;{\text{covered}}\;{\text{by}}\;{\text{regrowth}}\;{\text{hair}}\;\left( {{\text{cm}}^{2} } \right)}}{{4 \times 1.5\;\left( {{\text{cm}}^{2} } \right)\left( {{\text{Initial}}\;{\text{shaving}}\;{\text{area}}} \right)}} \times 100\% .$$

### Histological evaluation

The collected skin samples were fixed in 4% paraformaldehyde for 24 h and embedded in paraffin. To access the number change of hair follicles, 5 μm-thick cross-sections of the samples were prepared and stained with hematoxylin–eosin (HE). To access angiogenesis and hair follicles proliferation, sliced paraffin sections were immunohistochemically stained with anti-CD31(1:500 dilutions, ab182981, Abcam, Cambridge, UK) and anti-Ki67 (1:500 dilutions, ab16667, Abcam) respectively overnight at 4 °C. Then incubated with horseradish peroxidase-conjugated polymer anti-rabbit antibody (Dako, Denmark) for 1 h. The number of CD31-positive blood vessels and the percentage of Ki67-positive proliferating hair follicles cells were counted using an optical microscope (Nikon Corp., Japan) and obtained with the digital camera (Nikon DS-Ri2, Japan) and NIS-Elements AR 3.2 software. Images were 600 × 600 pixles with no downstream image processing and then analyzed with *Image J* (NIH *Image J* system, USA). At least three random fields were selected for each sample.

### Western blot analysis for tissues

To evaluate the expression of AR in vivo, approximately 2000 hair follicles were separated from the collected skin samples under Stereoscope (Olympus, Japan). Hair bulbs were then preserved and ground to powder in liquid nitrogen. Subsequently, the powder was incubated in clod RIPA buffer (Servicebio, China) and centrifuged at 16,000 rpm for 20 min at 4 °C to obtain total amount protein. Other steps were the same as a general western blot procedure for cell lysate. The primary antibodies used were Anti-AR (1:1000 dilution, AF6137, Affinity Biosciences, USA) and anti-β-actin (1:2000 dilution, ab8226, Abcam, USA).

### Cell culture and experimental design

Human dermal papilla cells (hDPCs) were purchased from ScienCell (California, USA) and cultured in Dulbecco' s Modified Eagles Medium-Nutrient Mixture F-12 (DMEM/F-12, Gibico, USA) containing 10% Fetal bovine serum (FBS, HyClone, USA) and 1% penicillin–streptomycin antibiotic (Gibico, USA) at 37 °C in a 5% CO2 environment. Media were changed every 5 days, and cells were used up to passages 4 for subsequent experiments.

In the following vitro study, we separate the experimental groups in 3 and each group contains 4 subgroups to evaluate the effect of CEFFE and DHT on hDPCs: Group^CEFFE^ (only treated with CEFFE), Group^DHT^ (only treated with DHT (Solarbio, China), Group^DHT+CEFFE^ (treated with CEFFE and DHT). The specific grouping method was shown in Table 1. Cells were observed and photographed by inverted phase-contrast microscopy (Nikon Eclipse TS100) with Nikon DXM1200 camera (Nikon, Japan) and NIS-Elements AR 3.2 software with 1290 × 1024 pixels without downstream image processing.

**Table 1:** In Group^CEFFE^, hDPCs were incubated with different concentrations of CEFFE (0, 50, 250, 500 μg/mL; 0 μg/mL CEFFE as control)

Groups	Name	Treatments
Group^CEFFE^	Group^CEFFE_0^	0 μg/mL CEFFE (Control)
	Group^CEFFE_50^	50 μg/mL CEFFE
	Group^CEFFE_250^	250 μg/mL CEFFE
	Group^CEFFE_500^	500 μg/mL CEFFE
Group^DHT^	Group^DHT_0^	0 μM DHT (Control)
	Group^DHT_1^	1 μM DHT
	Group^DHT_10^	10 μM DHT
	Group^DHT_100^	100 μM DHT
Group^DHT+CEFFE^	Group^DHT_100+CEFFE_0^	100 μM DHT + 0 μg/mL CEFFE (Control)
	Group^DHT_100+CEFFE_50^	100 μM DHT + 50 μg/mL CEFFE
	Group^DHT_100+CEFFE_250^	100 μM DHT + 250 μg/mL CEFFE
	Group^DHT_100+CEFFE_500^	100 μM DHT + 500 μg/mL CEFFE

In Group^DHT^, hDPCs were incubated with different concentrations of DHT (0, 1, 10, 100 μM; 0 μM DHT as control). In Group^DHT+CEFFE^, hDPCs were incubated with 100 μM DHT and different concentrations of CEFFE (0 + 100, 50 + 100, 250 + 100, 500 μg/mL + 100 μM; 0 μg/mL CEFFE + 100 μM DHT as control)

### Cell proliferation assay

Cell proliferation was evaluated by CCK-8 assay (Beyotime, China). hDPCs were seeded in 96-well plates (6 × 10^3^ cells per well) with a standard medium for 24 h and then treated with a fresh medium containing corresponding drug concentrations for another 72 h. Then the medium was replaced and incubated with CCK-8 reagents diluted in DMEM (1:9) for 2 h at 37 °C. The optical density value of each well was detected at a wavelength of 450 nm by a microplate reader (SpectraMAX190, Molecular Technologies, Japan).

### Cell cycle and apoptosis assays

Flow cytometry (Beckman Coulter, USA) was used for cell cycle and apoptosis assays. hDPCs were cultured in 6-well plates (2 × 10^5^ cells per well) for 24 h and treated as described above for either 48 h for cell cycle assay or 96 h for apoptosis assay. Then cells were harvested and washed with PBS 3 times. Cell cycle analysis kit (Beyotime, China) was used for cell cycle analysis, and Annexin V-FITV/PI apoptosis analysis kit (BD Biosciences, USA) was used for apoptosis assays following the manufacturer’s protocol. Results were analyzed by Kaluza Analysis Software v.2.0.

### Enzyme-linked immunosorbent assay

hDPCs were cultured in 6-well plates at a density of 4 × 10^5^ cells for 24 h, and the culture medium was changed as described above for another 72 h. After being lysed with RIPA (Servicebio, China), the lysis supernatant from different samples was collected and analyzed using a DHT Quantizing ELISA kit (DB52021, IBL, German) following the manufacturer’s protocol.

### Real-time quantitative-PCR (RT-qPCR)

hDPCs were seeded in 6-well plates (4 × 10^5^ cells per well) for 24 h and treated with corresponding drugs for 72 h. All cellular RNA was then extracted using Total RNA Extraction Reagent (EZBioscience, USA). 1 μg of the extracted RNA was reversed to cDNA using a reverse transcription master mix (EZBioscience, USA). Then RT-qPCR was conducted using an SYBR green qPCR master mix (ROX2 plus, EZBioscience, USA). The cycling parameters were 95 °C for 5 min, then 40 cycles at 95 °C for 10 s, followed by 60 °C for 30 s. At least three technical replicates were performed for each sample. Relative expression levels were calculated using the 2^−ΔΔCT^ method and were presented as fold-change compared to GAPDH expression. The primers for RT-qPCR are listed in Table 2.

**Table 2:** Primer pairs used in RT-qPCR

Gene names	Forward: 5′–3′	Reverse: 3′–5′
AR	CTCTCTCAAGAGTTTGGATGGCT	CACTTGCACAGAGATGATCTCTGC
SRD5A2	TTTCCTCGGTGAGATCATTGAATG	TTAGGTAGAACCTATGGTGGTG
AKR1C2	CCTATGCACCTCCAGAGGTTC	TTCGGATGGCCAGTCCAAC
AKR1C4	TCATGGAGAAGTGTAGGATGCAG	TCATGGAGAAGTGTAAGGATGCA
GAPDH	GGGAAGCTTGTCATCAATGGAA	AGAGATGACCCTTTTGGCTC

### Western blot analysis for cell

hDPCs were seeded in 10 cm plates at a density of 6 × 10^5^ cells for 24 h and then treated with various mediums for 72 h. Next, total proteins from different samples were obtained and quantified using a BCA assay. Equal amount of proteins was loaded and separated using 10% sodium dodecyl sulfate–polyacrylamide gel and transferred to polyvinylidene fluoride membranes. The membranes were then incubated with the primary and secondary antibodies (1:5000 dilution, 111-035-045, Jackson ImmunoResearch) and visualized using enhanced chemiluminescence (Pierce, USA). The primary antibodies used were Anti-AR (1:1000 dilution, AF6137, Affinity, USA), Anti-SRD5A2 (1:1000 dilution, Ab124877, Abcam, USA) and anti-β-actin (1:2000 dilution, Ab8226, Abcam, USA).

### Statistical analysis

All Data were presented as the mean ± standard deviation, and each value was averaged or calculated using three unbiased samples collected in the experimental data. Statistical analysis was performed using an independent-sample *t*-test or one-way analysis of variance, followed by Tukey’s post hoc test on SPSS 13.0 software (SPSS, Inc., Chicago, USA). A statistically significant difference was reported if *P* < 0.05.

## Results

### CEFFE contains low doses of sex hormones and is not immunogenic or genotoxic

The testosterone level in CEFFE was 0.18 ± 0.08 ng/mL, which was well below normal physiological levels (6.00–27.10 ng/mL), and the estradiol concentration in CEFFE was 0.02 ± 0.01 ng/mL, within the normal physiological range (0.011–0.044 ng/mL) (Additional file 2: Fig. S1A). Results of the in vitro cytotoxicity test showed that cell survival rates were 2%, 56%, 87%, and 125% for 100%, 50%, 25%, and 12.5% CEFFE, respectively, with potential cytotoxicity at CEFFE volume fractions > 25% (Additional file 2: Fig. S1B). In the intracutaneous reactivity test, no erythema and edema was observed in each animals in 24, 48, 72 h after injection of CEFFE, and the difference between the mean scores of the CEFFE experimental group and the blank control group was < 1 (Additional file 1: Table S2). In the system in toxicity assay, no signs of systemic toxicity such as weight loss or death were observed in CEFFE experimental group (Additional file 1: Tables S3, S4). In the pyrogen test, the increase in body temperature in three rabbits was 0 °C, 0 °C and 0 °C respectively. The combined increase in body temperature was 0 °C, which satisfied the criteria of < 0.6 °C for each rabbit or < 1.3 °C for the total increase in body temperature. CEFFE was judged to have no pyrogenic effect. The above experiments were combined to determine that CEFFE was non-immunogenic. In Ames assay, the negative control and the positive control met the criteria. CEFFE did not increase the number of colonies of 4 strains by twofold or more compared with the negative control (Additional file 1: Table S5). In MLA assay, the negative control and the positive control met the criteria, and the frequency of CEFFE induced mutations in the TK gene was similar to that of the negative control (Additional file 1: Table S6). The results of these two experiments showed that CEFFE was non-genotoxic.

### CEFFE reversed hair regrowth delay induced by DHT through blocking AR

During the experiment, no adverse health events were recorede. To determine whether CEFFE could promote hair regrowth in DHT-induced AGA mice models, consecutively taken photographs on shaved area was evaluated. Skin color, anagen entry rate (%) and hair coverage percentage (%) for different groups were compared and calculated as described previously. All mice were depilated and start from telogen phase to eliminate inter-subject differences and allow synchronous hair regeneration. Normally, the shaved skin of C57BL/6mice was pink during the telogen phase and darkens as entering anagen phase. As shown in Fig. 1C, seven days after depilation, the skin of control mice was pigmented (anagen phase), whereas Model group mice was still pink until D10, indicating a significant delay in the reentry of the hair follicles into the anagen phase. However, in the CEFFE treatment groups, the skin color changed from pink to grey on D8, D6, and D8 respectively for CEFFE^Low^, CEFFE^Middle^, and CEFFE^High^ groups, which aremuch earlier than the Model group (Fig. 1D). By D14, the CEFFE treatment group displayed more than 60% hair regrowth, reached more than 90% hair regrowth after D16. (Fig. 1E) The hair regrowth of the CEFFE treatment group showed statistical significance compared to the Model group with *P* < 0.05.

### CEFFE promoted DPCs proliferation and enhanced angiogenesis

**Fig. 2:**
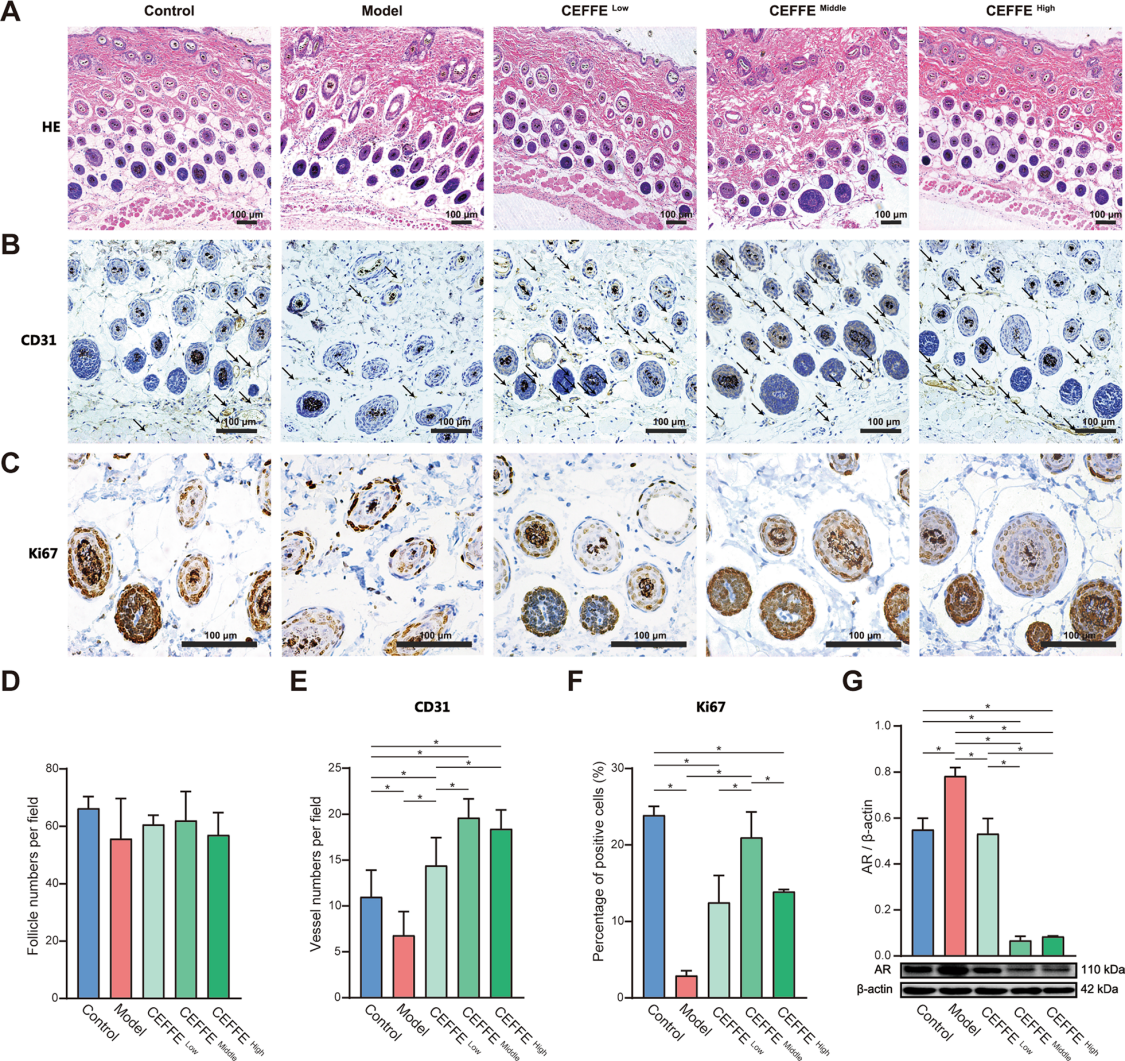
Sagittal sectional views of histological observation of hair follicles in C57BL/6 mice. **A** HE staining on dorsal skin taken from the shaved area with 4 × objective lens. Scale bar = 100 μm. **B** Quantitative analysis on the number of hair follicles. **C** CD31 immuno-histochemical staining of dermal sections with 10 × objective lens (black arrows indicate CD31—positive micro-vessels). Scale bar = 100 μm. **D** Quantitative analysis of CD31 staining surrounding hair follicles. **E** Ki67 immunohistochemically staining of dermal sections with 20 × objective lens. Scale bar = 100 μm. **F** Quantitative analysis of Ki67 staining inside hair follicles. **G** Western blot analysis probing for androgen receptor (AR) after CEFFE treatment. Density values were presented in arbitrary units (AU). Original data was presented in Additional file 2: Fig. S3. Data represent the mean ± SD; *P < 0.05

To investigate DHT-induced histological changes, dorsal skin samples were taken from all subjects and studied through HE staining and immunohistochemistry for proliferation marker Ki67 and angiogenesis marker CD31 (Fig. 2A–C). The number of hair follicles per field was counted quantitatively, and the morphology of the inner and out root sheath of hair was displayed. There was no significant difference in HF number and morphology among groups (*P* > 0.05), which might be due to a too short DHT modeling time (Fig. 2D). The immunohistochemical stain of CD31 showed that the number of blood vessels per field surrounding dermal papillae was significantly decreased in the Model group (6.8 ± 2.59) compared with the control group (11.0 ± 2.92) (*P* < 0.05). The number of CD31-positive blood vessels in all CEFFE treated groups (14.4 ± 3.04 for CEFFE^Low^; 19.61 ± 2.07 for CEFFE^Middle^; 18.4 ± 2.08 for CEFFE^High^) was much higher compared to the Model group (*P* < 0.05), with the highest in the CEFFE^Middle^ group (Fig. 2E). The immunohistochemical stain of Ki67 showed a decrease in Ki67-positive proliferation cells for Model mice (3.38 ± 0.76%), compared to the control group 23.93 ± 0.98% (*P* < 0.05). However, in the CEFFE treatment group, the number of Ki67-positive increased sharply after being treated with CEFFE (Fig. 2F). 16.17 ± 11.89% for CEFFE^Low^; 24.84 ± 20.22% for CEFFE^Middle^; 14.79 ± 13.86% for CEFFE^High^ (*P* < 0.05) respectively. Among three treatment groups, CEFFE^Middle^ showed the best proliferation promotion ability.

### CEFFE decreased the DHT-induced high expression of AR

To evaluate the regulation effect of CEFFE on AR expression in vivo, western blot probing for AR in hair bulbs was performed. Results showed that the expression of AR in the Model group was much higher than in the control group (*P* < 0.05), indicating DHT upregulate AR expression. In contrast, the expression of AR was decreased in the CEFFE treatment group compared to the Model group, and CEFFE^Middle^ group showed most robust inhibition of AR protein expression (Fig. 2G). Therefore, CEFFE^Middle^ may be the optimal dosing regimen to cure DHT-induced AGA in this study.

### CEFFE reversed decrease in proliferation and abnormal cell cycle arrest of hDPCs caused by DHT

**Fig. 3:**
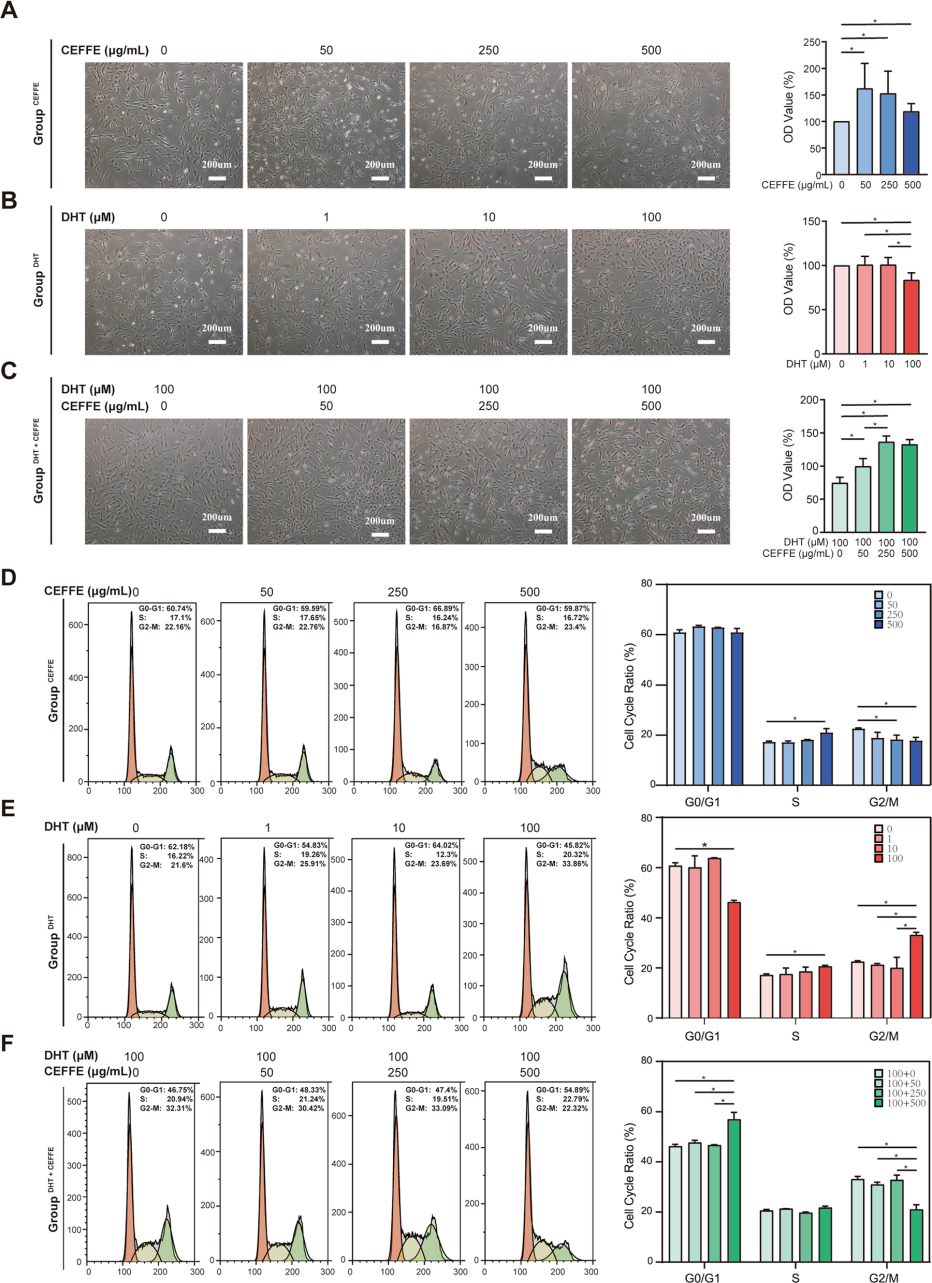
Effect of CEFFE (0, 50, 250, 500 μg/ml; 72 h), DHT (0, 1, 10, 100 μM; 72 h) and DHT + CEFFE (100 μM DHT + 0, 50, 250, 500 μg/ml CEFFE; 72 h) on Human dermal papilla cells (hDPCs) proliferation and cell cycle. **A**–**C** Morphology of hDPCs in Group^CEFFE^, Group^DHT^ and Group^DHT+CEFFE^ and its relevant quantitative analysis of hDPCs proliferation detected by the CCK-8 assay among different groups. **D**–**F** hDPCs cell cycle distribution analyzed by flow cytometry in Group^CEFFE^, Group^DHT^ and Group^DHT+CEFFE^ and their quantitative analysis of cell cycle distribution. Scale bar = 200 μm; Data represent the mean ± SD; *n* = 3; *P < 0.05

To investigate the influence of CEFFE on hDPCs proliferation with or without DHT, direct observation using light microscopic and CCK-8 assay were applied to different groups. In Group^CEFFE^, CEFFE showed a consistent ability to promote cell proliferation regardless of CEFFE concentration. The optimal proliferation of hDPCs was detected at CEFFE concentration of 50 μg/mL (Fig. 3A). In Group^DHT^, fewer cells were observed following the increase in concentration of DHT treatment, indicating a stronger proliferation inhibition (Fig. 3B). Therefore, 100 μM DHT was used as the modeling concentration to simulate the high DHT concentration in the patients with AGA. For Group^DHT+CEFFE^, results showed that CEFFE rescued hDPCs from DHT (100 μM)- related stress. The 250 μg/mL of CEFFE treatment among all different concentrations has the best therapeutic effects (Fig. 3C).

To further explore potential mechanisms of the growth-promoting effect of CEFFE on hDPCs with and without DHT, cell-cycle and apoptosis analysis were performed using flow cytometry. In Group^CEFFE^ (Fig. 3D), CEFFE led to a reduce amount of cells in G2/M- phase and an increase amount of cells in S-phase comparing to control subgroup (*P* < 0.05). On the contrary, in Group^DHT^, there was a significant increase (*P* < 0.05) of hDPCs in G2/M phases as compared to the untreated cells, with 100 μM DHT showed the most substantial G2/M arrest effect (Fig. 3E). In Group^DHT+CEFFE^, CEFFE showed the ability to reverse cell cycle arrest at the G2/M phase induced by 100 μM DHT in a dose-dependent manner and showed the best effect at 500 μg/mL(Fig. 3F).

### CEFFE inhibited apoptosis induced by DHT in hDPCs

**Fig. 4:**
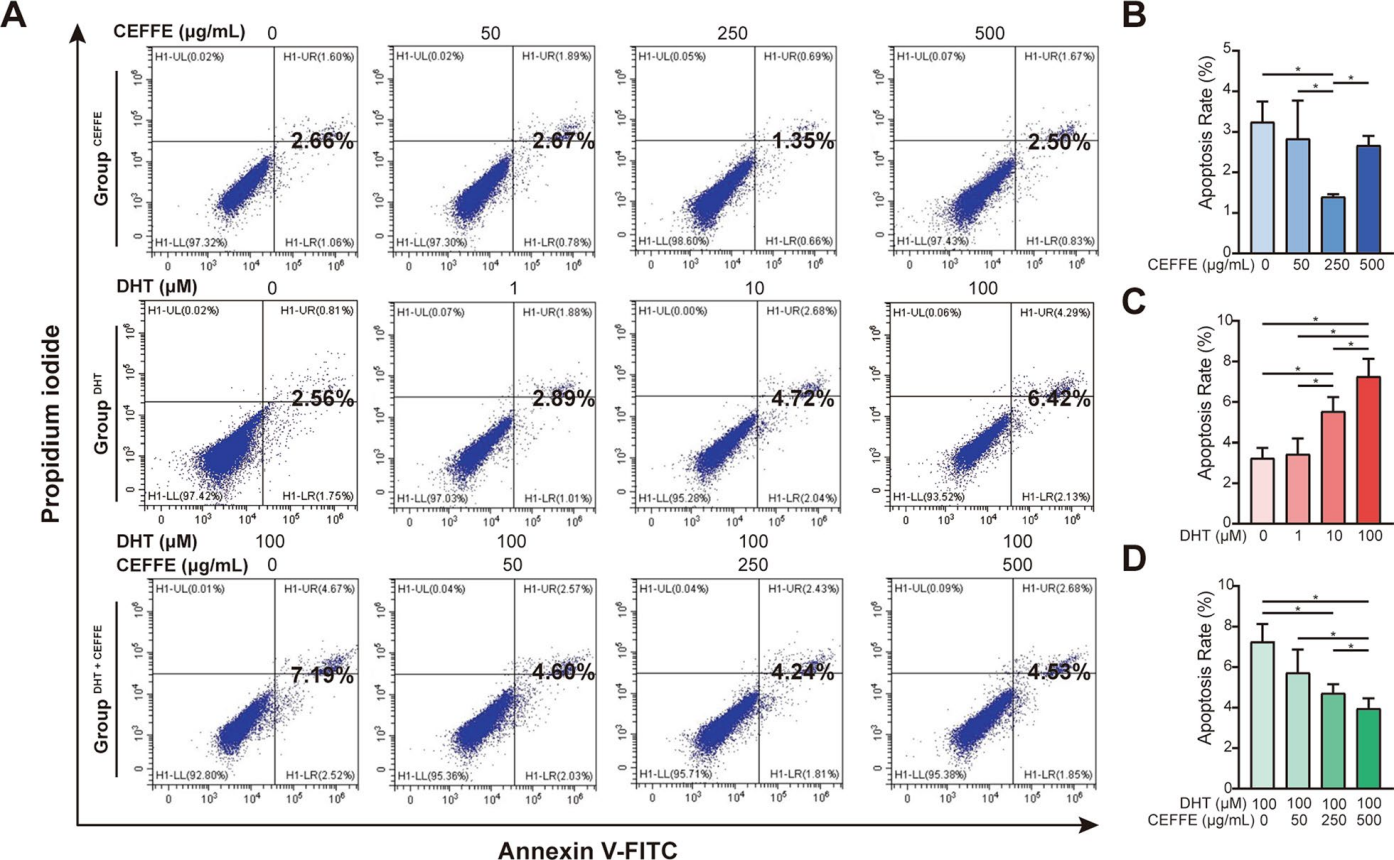
Effect of CEFFE (0, 50, 250, 500 μg/ml; 72 h), DHT (0, 1, 10, 100 μM; 72 h) and DHT + CEFFE (100 μM DHT + 0, 50, 250, 500 μg/ml CEFFE; 72 h) on cell apoptosis. **A** Effect of CEFFE, DHT and DHT + CEFFE on hDPCs apoptosis was assessed with flow cytometry with Annexin V-FITC/PI staining. **B**–**D** Quantitative analysis of the cell apoptosis assay among different groups. Data represent the mean ± SD; *n* = 3; *P < 0.05

In apoptosis analysis, early apoptotic cells (Annexin V- positive, PI-negative) and late apoptotic cells (Annexin V- positive, PI-positive) were evaluated (Fig. 4A). As Fig. 4B showed, the amount of apoptotic hDPCs was significantly reduced when treated with 250 μg/mL CEFFE in Group^CEFFE^(*P* < 0.05). In contrast, a dosage-dependent apoptotic increase was observed upon DHT treatment in Group^DHT^ (Fig. 4C) (*P* < 0.05). In Group^DHT+CEFFE^ (Fig. 4D), CEFFE was shown to inhibit apoptosis induced by DHT by affecting DHT-induced G2/M cell cycle arrest (*P* < 0.05).

### CEFFE decreased intracellular DHT concentration and reduced AR expression in hDPCs

**Fig. 5:**
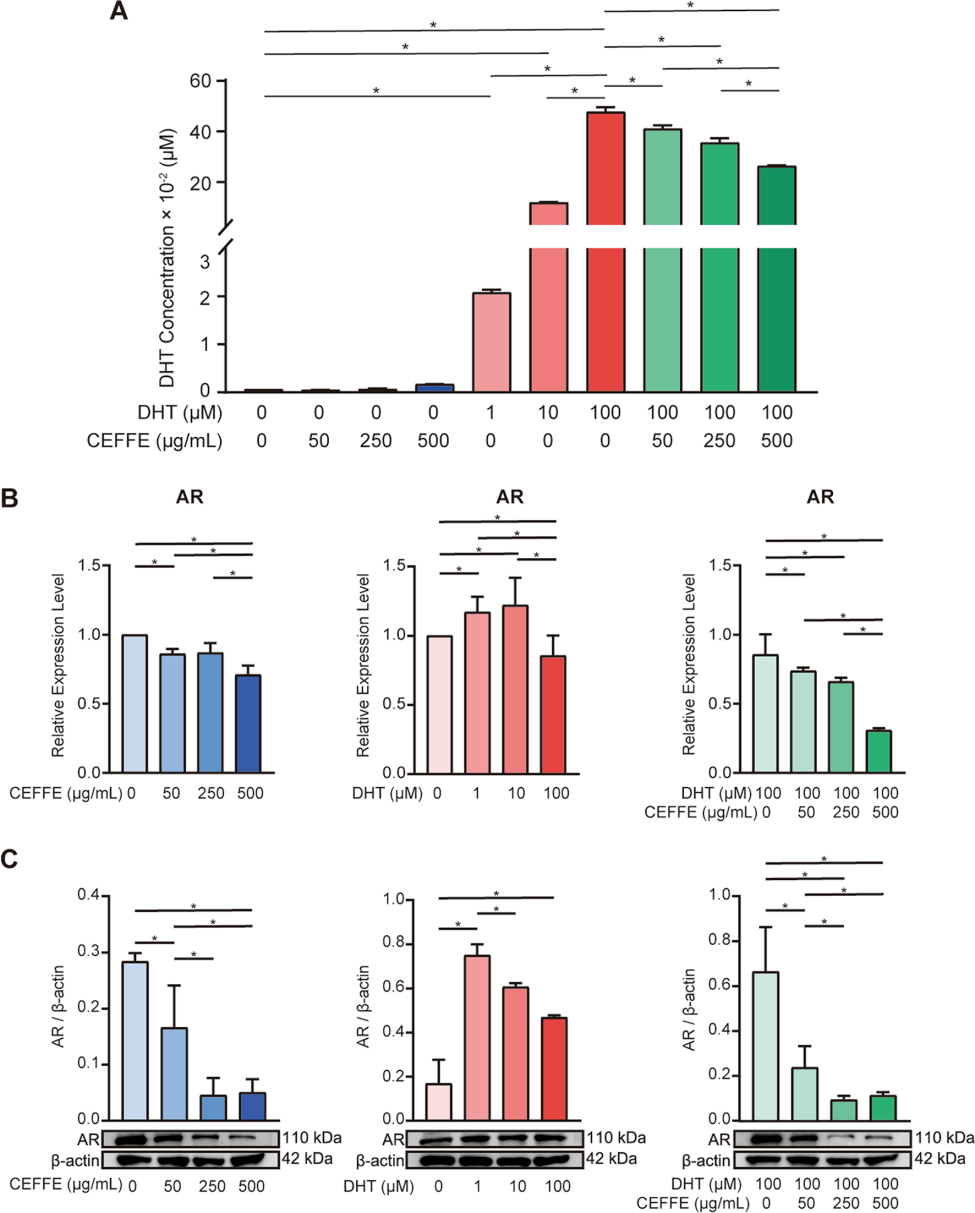
Effect of CEFFE (0, 50, 250, 500 μg/ml; 72 h), DHT (1, 10, 100 μM; 72 h) and DHT + CEFFE (100 μM DHT + 50, 250, 500 μg/ml CEFFE; 72 h) on the intracellular DHT concentration and AR expression of hDPCs. **A** Quantitative analysis of DHT concentration using ELISA. **B** RT-qPCR analysis of AR mRNA expression after culturing hDPCs with CEFFE, DHT and DHT + CEFFE, respectively. qRT-PCR analysis showed the expression of AR was increased after DHT treatment but declined after co-incubation with DHT and CEFFE. **C** Western blot for AR after treating hDPCs with CEFFE, DHT and DHT + CEFFE. Original data was presented in Additional file 2: Fig. 4. It revealed an increase in the expression of AR after incubating with DHT but decreased after co-incubation with DHT and CEFFE. Data represent the mean ± SD; *n* = 3; *P < 0.05

An ELISA assay was applied to evaluate the effect of CEFFE and DHT on intracellular DHT concentration. In Group^CEFFE^ and Group^DHT^, while the concentration of intracellular DHT showed a rising trend, the maximal change in Group^DHT^ varied over five orders of magnitude, and the maximal variation in Group^CEFFE^ was only two orders of magnitude. In Group^DHT+CEFFE^, the intracellular DHT concentration decreases as CEFFE increase in concentration (Fig. 5A). Meanwhile, we have test DHT production-related enzymes and metabolism-related enzymes. In the absence of DHT, 250 and 500 μg/mL of CEFFE caused a decrease in 5α-reductase type II (SRD5A2) expression in hDPCs (*P* < 0.05, Additional file 2: Figs. S2A, B, S5). The expression of enzymes associated with DHT metabolism (AKR1C2 and AKR1C4) were significantly increased in the presence or absence of DHT, with the most pronounced effect at 500 μg/mL of CEFFE (*P* < 0.05, Additional file 2: Fig. 2C, D).

Besides ELISA, in order to evaluate the expression level of AR in hDPCs cells, RT-qPCR and Western blot were performed for further analysis. As results showed, DHT significantly increased the mRNA concentrations (Fig. 5B) and protein expressions of AR (Fig. 5C) in Group^DHT^ compared to control. However, the AR expression was remarkably inhibited by CEFFE in a dose-dependent manner with or even without DHT treatment, suggesting CEFFE inhibit AR signaling pathway by suppressing the expression of the AR gene and corresponding AR protein synthesis. This result was consistent with vitro experimental results.

## Discussion

AGA is the most common hair disorder and thus requires more satisfactory treatments. Recent advances in regenerative therapies, including stem cells and platelet-rich plasma, which mainly depend on bioactive factors, have raised new hopes and paved the way for new therapies against hair loss [35–38]. However, concerns about the biosafety of those biological therapies have hindered their clinical application. CEFFE, which contains similar bioactive factors to stem cells' paracrine composition, was raised as a cell-free strategy to overcome these challenges [24, 39]. In the current study, we proved the safety and therapeutic effect of CEFFE and provided multilevel biological evidence to unravel the underlying mechanism in vivo (DHT-induced mice) and in vitro (hDPCs).

Hormones should be considered when using CEFFE. We have detected testosterone, dihydrotestosterone, estradiol and progesterone levels in CEFFE from 20 female samples via ELISA test. The results showed both of the testosterone and dihydrotestosterone were undetectable and the progesterone level was barely detectable. We have also tested hormone levels in male samples, which indicated that testosterone concentration was very low and the estradiol levels with normal range. It can be presumed that hormone levels in CEFFE have no effect on AGA treatment. Meanwhile, we have also tested the immunogenicity and genotoxicity of CEFFE, and combined results prove the safety of CEFFE due to its cell-free characteristics.

Hair follicles in AGA patients were remodeled during cyclical periods of growth (anagen), regression (catagen), and rest (telogen) [40, 41]. The length of the rapid growth stage of anagen mainly contributes to hair growth. Therefore, maintaining the anagen cycle was vital for the treatment of AGA. Our results confirmed that, in DHT-induced AGA mice models, the color change of mice skin from pink to grey was later, and the hair coverage percentage was lower than in the control group. Those phenomena indicated that DHT could short catagen phases and slow down the rate of hair growth. On the other hand, we observed that CEFFE treatment could reverse this situation, presenting as the extension of anagen phased and the acceleration hair growth rate. Moreover, we observed that CEFFE^Middle^-frequency might have a better therapeutic effect than other CEFFE treatment groups.

Previous studies show that DPCs in hair follicles regulates the hair cycle [42]. Specifically, in DPCs, DHT was bound to AR, and the hormone-receptor complex activates the target gene to regulate anagen-to-catagen transition [43]. Thus, the direct influence on the state of DPCs or the indirect influence on DHT/AR may influence the progression of AGA. In vivo study, although the number and the morphology of mouse hair follicles in the Model group showed no significant difference from the Control group, the quantification of Ki67-positive cells showed a significant decline. However, this trend could be reversed by CEFFE treatment, especially in the CEFFE^Middle^ group, presenting as the protective effect of hair follicles cells. In addition, the result of the in vitro experiments was consistent with that of the in vivo experiment. In vitro study, DHT reduced the proliferation of hDPCs in a dose-dependent manner, and 100 μM of DHT showed the most potent inhibition, the same as previously reported [44].

Moreover, we observed promotion in the proliferation of hDPCs after culturing with CEFFE regardless of the presence or absence of DHT. In other studies, HGF was associated with the span of the hair follicle stages, and platelet-derived growth factor (PDGF) promotes and maintains the anagen stage. Thus, we speculate that HGF and PDGF may play a crucial role in hair cycle control [45–47]. Furthermore, we also demonstrated that the promotion effect of CEFFE on hDPCs proliferation might be due to the inhibition of cell cycle arrest and apoptosis. In Group^DHT^, we found that DHT induced G2/M cell cycle arrest and increased the apoptotic rate of hDPCs. In contrast, the G2/M cell cycle arrest was abrogated, and the cell apoptosis rate decreased after culturing with CEFFE in Group^CFEEF^ as well as Group^DHT+CEFFE^.

At the level of mechanism study, we considered that CEFFE could exert a therapeutic role by mediating DHT/AR pathway. In vitro study, the result of ELISA showed that intracellular concentration of DHT was significantly increased after culturing hDPCs with DHT, which was almost 20 times larger than that without DHT treatment. On the contrary, in Group^DHT+CEFFE^, the intracellular concentration of DHT declined in a dose-dependent manner. This phenomenon may be related to the decline of 5α-reductase type II expression, which was related to DHT production. Meanwhile, the elevated expression of DHT-degrading enzymes in hDPCs may be also favorable. There was a study confirmed that Sulforaphane increases the expression of DHT-degrading enzymes to treat AGA in mice [48]. Besides, RT-qPCR and western blot proved that CEFFE could reduce the expression of AR with or without DHT, which may block the effect of AR and DHT [49]. Furthermore, in vivo study, the result of tissue western blot also confirmed that CEFFE could inhibit AR expression in DHT-induced mice, consistent with results from cell experiments. According to previous studies, IGF-1 could play an essential role in this process either directly via the reduction of extracellular concentration of DHT or indirectly via the inhibition of AR expression [50].

Another issue of hair loss was the insufficiency of blood supplements. Hair loss symptoms were relieved when promoting angiogenesis, such as injecting platelet-rich plasma or low-level laser therapy [51–53]. As our CD-31 immunohistochemically staining showed, DHT reduced vessels number of mice in the model group compared with the standard group. On the contrary, after treating with CEFFE, the number of CD-31 positive angiogenesis per field was significantly increased, which suggested that CEFFE could stimulate neovascularization in the presence of DHT. Increasing angiogenesis enhanced blood supply for the skin and scalp and accelerated the metabolism of DHT to reduce its impact. According to other studies, VEGF may promote angiogenesis in mice [45].

Taken together, CEFEE showed a therapeutic effect on AGA in both in vivo and in vitro models. The specific mechanism is still unclear, but it is likely due to a combination of the protection of DPCs, the regulation of DHT/AR, and the promotion of neovascularization [54]. There are certain promising prospects of CEFFE, but its application in clinical needs further in-depth study. First of all, although some factors in CEFFE were well-documented to be correlated with hair regrowth, the specific component of CEFFE still stayed unclear. There were also some factors harmful for the treatment of hair loss, which have been observed in the cell culture study that higher concentration of CEFFE did not show better results (Fig. 3A). Therefore, the specific active ingredients and the optimal treatment dose of CEFFE in clinical treatment remains to be determined.

In addition, the preparation of CEFFE was rapid, simple, convenient and straightforward with its advantage of not using any exogenous agents, and only once preparation could meet clinical needs. Excess CEFFE could be stored conveniently at -80 °C for future long-term AGA treatment. The clinical application of CEFFE is similar to ADSCs-conditioned media (ADSC-CM), which is also a cell-free liquid. Subcutaneous injection or application with micro-needling weekly of ADSC-CM for 12 weeks was demonstrated as optimal method in treating AGA, which was also an option for CEFFE [39].

As for the quality control of CEFFE from different donors, donor's age, sex, and chronic disease would impact the composition of growth factors in CEFFE, especially the chronic diseases [55, 56]. CEFFE from elder people and male have not been analyzed before. In our previous study, the concentrations of major growth factors in CEFFE from healthy young females with different age (22 to 35) and different aspiration site were relatively stable [24]. Thus, we believe that allogenic CEFFE would be an ideal source for those patients. What’s more, a combination of CEFFE with existing drugs, such as Minoxidil and Finasteride, might work better. Comparing the efficacy of CEFFE, commercially available drug, and the combination of them in treating AGA is worth to be tested in the animal studies as well as in the future clinical trials.

Therefore, CEFFE appears a potential therapeutic option for AGA treatment due to its various bioactive factors composition, the absence of immunogenic properties, the simplicity of acquisition, and the simplicity of separation.

## Conclusion

**Fig. 6:**
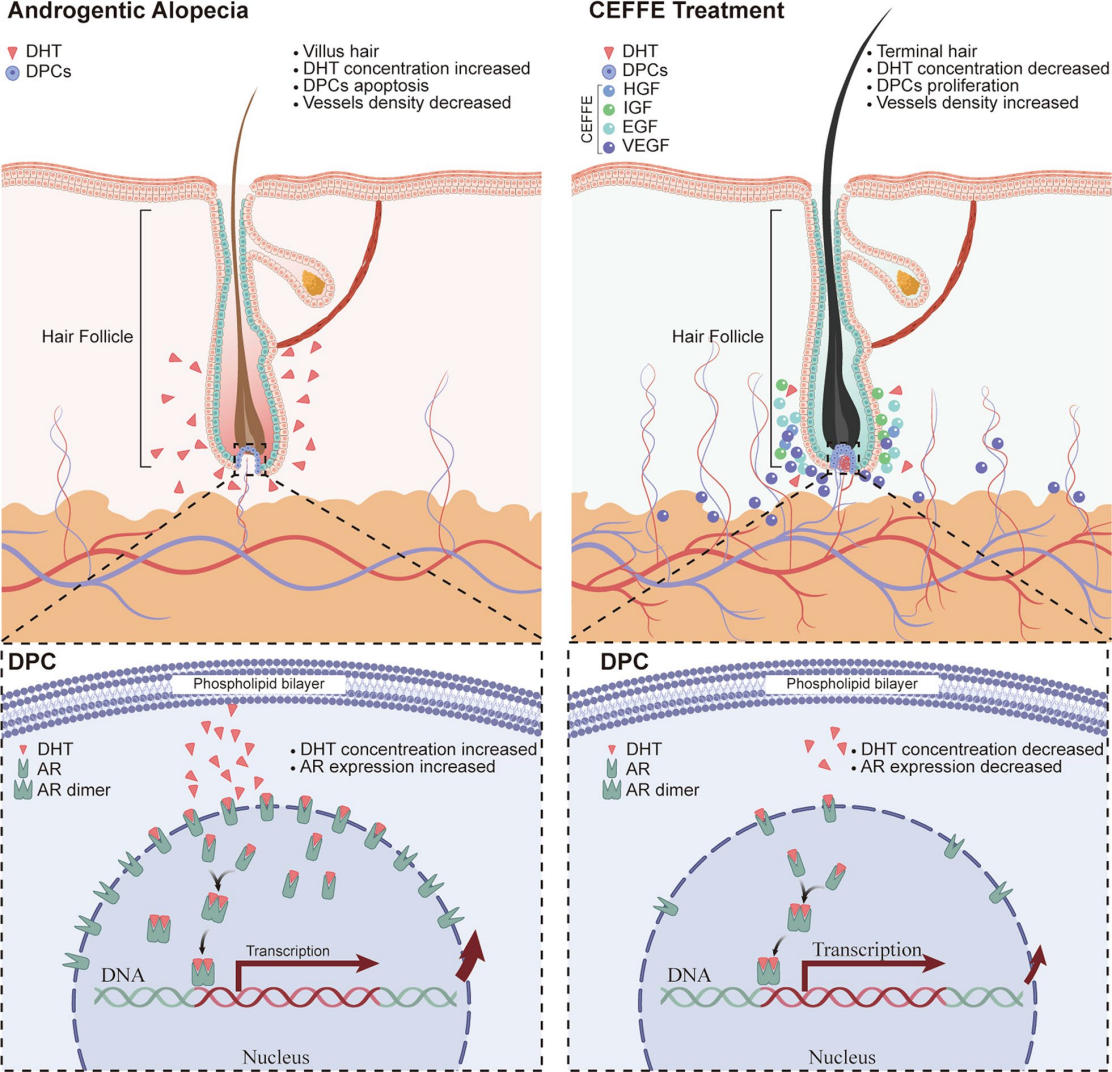
CEFFE promote hair growth in AGA via mediating DHT/AR pathway in DPC to enhance cell proliferation, modulate the cell cycle, and increase neovascularization in the DHT-induced model

This study demonstrated that CEFFE promoted hair growth in AGA via mediating DHT/AR pathway in DPC to enhance cell proliferation, modulate the cell cycle, and increase neovascularization in the DHT-induced model (Fig. 6). Furthermore, anti-inflammation might be another potential mechanism for treating AGA. cell-free feature of CEFFE makes it safe to use without the risk of immunogenicity and genotoxicity. Thus, CEFFE could potentially be a novel and effective therapeutic strategy for AGA.

### Supplementary Information

**Additional file 1: Table S1.** Patient information. **Table S2.** Results of the intracutaneous reactivity test. **Table S3.** Clinical signs/observations to systemic toxicity assay. **Table S4.** Variation in the weight of the animal in systemic toxicity assay. **Table S5.** Results of Ames test. **Table S6.** Results of MLA assay.

**Additional file 2: Figure S1.** Hormone levels evaluation and in vitro cytotoxicity test results. **Figure S2**. RT-qPCR or/and western blot analysis of DHT-producing enzymes SRD5A2 and DHT-degrading enzymesin hDPCs co-cultured with CEFFE 72 h. **Figure S3.** Original data of AR western blot results in hair follicles of C57BL/6 mice. **Figure S4.** Original data of AR western blot results in hDPCs. **Figure S5.** Original data of SRD5A2 western blot results in hDPCs  .

## Data Availability

The datasets during the current study are available from the corresponding author on reasonable request.

## Abbreviations

AGA: Androgenetic alopecia
ADSCs: Adipose-derived stem cells
ANOVA: Analysis of variance
AR: Androgen receptor
CEFFE: Cell-free fat extract
CM: Conditioned media
DHT: Dihydrotestosterone
DPCs: Dermal papilla cells
ELISA: Enzyme-linked immunosorbent assay
hDPCs: Human dermal papilla cells
HE: Hematoxylin–eosin
HGF: Hepatocyte growth factor
IGF: Insulin-like growth factor
MLA assay: Mouse lymohoma TK assay
PDGF: Platelet-derived growth factor
PG: Prostaglandin
PRP: Platelet-rich plasma
RT-qPCR: Reverse transcription-quantitative polymerase chain reaction
VEGF: Vascular endothelial growth factor
