# Supplementary material for: Cell-free fat extract restores hair loss: a novel therapeutic strategy for androgenetic alopecia
Source: Stem Cell Res Ther. 2023 Aug 23;14:219. doi: 10.1186/s13287-023-03398-1 (PMC10464375; doi:10.1186/s13287-023-03398-1)
Supplement: Supplementary file 1 — Additional file 1: Table S1. Patient information. Table S2. Results of the intracutaneous reactivity test. Table S3. Clinical signs/observations to systemic toxicity assay. Table S4. Variation in the weight of the animal in systemic toxicity assay. Table S5. Results of Ames test. Table S6. Results of MLA assay. [file 13287_2023_3398_MOESM1_ESM.docx]

# *Title:* Cell-free fat extract restores hair loss: a novel therapeutic strategy for androgenetic alopecia

***Authors:*** Yizuo Cai ^1, †^, Zhuoxuan Jia ^1, †^, Yichen Zhang ^2^, Bijun Kang ^1^, Chingyu Chen ^1^, Wei Liu ^1, *^, Wei Li ^1, *^, and Wenjie Zhang ^1, *^

***Institutions:*** ^1^ Department of Plastic and Reconstructive Surgery, Shanghai 9th People's Hospital, Shanghai Jiao Tong University School of Medicine, Shanghai Key Laboratory of Tissue Engineering, National Tissue Engineering Center of China, 639 ZhiZaoJu Road, Shanghai 200011, China; ^2^ Department of Biological and Environmental Engineering, Cornell University

***Co-Author:*** Yizuo Cai; Zhuoxuan Jia

***Corresponding Author****:* ^*^Wei Liu (Email: liuwei_md@126.com); ^*^Wei Li (Email: [liweiboshi@163.com](mailto:liweiboshi@163.com)); ^*^Wenjie Zhang (Email: [wenjieboshi@aliyun.com](mailto:wenjieboshi@aliyun.com))

* Correspondence: [liweiboshi@163.com](mailto:liweiboshi@163.com) (Wei Li); [wenjieboshi@aliyun.com](mailto:wenjieboshi@aliyun.com) (Wenjie Zhang)

| Patients no. | Sex | Age (year) | Height (cm) | Weight (kg) | BMI^1^ |
| --- | --- | --- | --- | --- | --- |
| 1 | Male | 42 | 168 | 73 | 25.86 |
| 2 | Male | 28 | 176 | 79 | 25.50 |
| 3 | Male | 36 | 175 | 72 | 23.51 |
| 4 | Male | 34 | 181 | 92 | 28.08 |
| 5 | Male | 38 | 178 | 78 | 24.62 |
| 6 | Male | 45 | 183 | 89 | 26.58 |
| 7 | Male | 43 | 172 | 75 | 25.35 |
| 8 | Male | 37 | 176 | 74 | 23.89 |

**Supplementary table 1.** Patient information. 1. BMI: body mass index.

|  |  | Animal No.1 | | Animal No.2 | | Animal No.3 | |
| --- | --- | --- | --- | --- | --- | --- | --- |
|  |  | EG^1^ | CG^2^ | EG | CG | EG | CG |
| 24 hours | Erythema | 0 | 0 | 0 | 0 | 0 | 0 |
|  | Edema | 0 | 0 | 0 | 0 | 0 | 0 |
| 48 hours | Erythema | 0 | 0 | 0 | 0 | 0 | 0 |
|  | Edema | 0 | 0 | 0 | 0 | 0 | 0 |
| 72 hours | Erythema | 0 | 0 | 0 | 0 | 0 | 0 |
|  | Edema | 0 | 0 | 0 | 0 | 0 | 0 |
| Average of 3 time points (Erythema + Edema) | | 0 | 0 | 0 | 0 | 0 | 0 |
| Average of all EG sites (A) = 0; Average of all CG sites (B) = 0 | | | | | | | |
| Final score of intra-cutaneous reactivity test (A - B) = 0 | | | | | | | |

**Supple****mentary table 2.** Results of the intracutaneous reactivity test. 1. EG: experimental group (0.2 mL 0.9% NaCl); 2. CG: control group (0.2 mL CEFFE);

| Group | Animal No. | Clinical evaluation | | | | | Mortality |
| --- | --- | --- | --- | --- | --- | --- | --- |
|  |  | Immediately  post injection | 4 hours | 24 hours | 48 hours | 72 hours |  |
| EG^1^ | 1 | NO^3^ | NO | NO | NO | NO | 0/5 (0%) |
|  | 2 | NO | NO | NO | NO | NO |  |
|  | 3 | NO | NO | NO | NO | NO |  |
|  | 4 | NO | NO | NO | NO | NO |  |
|  | 5 | NO | NO | NO | NO | NO |  |
| CG^2^ | 1 | NO | NO | NO | NO | NO | 0/5 (0%) |
|  | 2 | NO | NO | NO | NO | NO |  |
|  | 3 | NO | NO | NO | NO | NO |  |
|  | 4 | NO | NO | NO | NO | NO |  |
|  | 5 | NO | NO | NO | NO | NO |  |

**Supplementary t****able 3.** Clinical signs/observations to systemic toxicity assay. 1. EG: experimental group (50 ml/kg CEFFE); 2. CG: control group (50 ml/kg 0.9% NaCl); 3. NO: no observation.

| Group | Immediately  post injection weight (g) | 24 hours weight (g) | 48 hours weight (g) | 72 hours weight (g) |
| --- | --- | --- | --- | --- |
| EG | 20.12 ± 0.98  (N^1^ = 5) | 22.58 ± 1.03  (N = 5) | 24.90 ± 1.45  (N = 5) | 27.56 ± 1.62  (N = 5) |
| CG | 20.10 ± 0.34  (N = 5) | 22.84 ± 0.47  (N = 5) | 25.46 ± 0.96  (N = 5) | 28.02 ± 1.16  (N = 5) |
| P value | 0.97 | 0.62 | 0.49 | 0.62 |

**Supplementary table 4.** Variation in the weight of the animal in systemic toxicity assay. 1. N: Number of animals in the group

| Group | S9 | TA97 | TA98 | TA100 | TA102 | Results (compare with NG) |
| --- | --- | --- | --- | --- | --- | --- |
| NG^3^ | -^1^ | 114 | 39 | 144 | 256 | - |
|  | +^2^ | 103 | 43 | 147 | 271 |  |
| PG^4^ | - | 2021 | 942 | 1006 | 2061 | ± S9: Positive |
|  | + | 2650 | 961 | 887 | 2835 |  |
| EG^5^ | - | 113 | 41 | 137 | 268 | ± S9: Negative |
|  | + | 103 | 38 | 140 | 274 |  |

**Supplementary** **table 5.** Results of Ames test. 1. - S9:0.5 mL phosphate buffer solution; 2. + S9: 0.5mL S9 solution; 3. NG: negative group (0.1 mL 0.9% NaCl); 4. PG: positive group (0.1 mL fenaminosulf for -S9 TA97, TA98 and TA100; 0.1 mL Methyl Methane sulfonate for -S9 TA102; 0.1 mL fenaminosulf for +S9 TA97 and TA98; 0.1 mL 2-Aceetylaminofluorene for +S9 TA100; 0.1 mL Methyl Methane sulfonate for +S9 TA102); 5. EG: experimental group (0.1 mL CEFFE)

| Group | S9 | PE_0_ | RS (%) | PE_2_ | T-MF (× 10^-6^) | S-MF (× 10^-6^) | Determination criteria | Results （compare with NG） |
| --- | --- | --- | --- | --- | --- | --- | --- | --- |
| NG^3^ | -^1^ | 0.77 | 100.00 | 0.83 | 82.40 | 40.60 | 0.65 – 1.2 | - |
|  | +^2^ | 0.70 | 100.00 | 0.67 | 91.20 | 38.00 |  |  |
| PG^4^ | - | 0.61 | 79.60 | 0.50 | 459.90 | 245.90 | (1) 300 × 10^-6^ of T-MF higher than NG; or (2) 120 × 10^-6^ of S-MF higher than NG | ± S9: Positive |
|  | + | 0.28 | 40.70 | 0.33 | 773.50 | 382.60 |  |  |
| EG^5^ | - | 0.65 | 84.20 | 0.49 | 32.60 | 16.10 | (1) 126 × 10^-6^ of T-MF higher than NG; and (2) RS > 20% | ± S9: Negative |
|  | + | 0.77 | 110.60 | 0.66 | 110.60 | 57.50 |  |  |

**Supplementary table** **6**. Results of MLA assay. 1. - S9: 1 mL 150 mmol/L potassium chloride solution; 2. + S9: 1mL S9 solution prepared from rat liver; 3. NG: negative group (9 mL RPMI medium); 4. PG: positive group (0.2 mL 10 μg/mL MMS + 8.8 mL RPMI medium for - S9; 0.2 mL 3μg/mL CTX + 8.8 mL RPMI medium for + S9); 5. EG: experimental group (9 mL CEFFE)
