# Supplementary material for: Cell-free fat extract restores hair loss: a novel therapeutic strategy for androgenetic alopecia
Source: Stem Cell Res Ther. 2023 Aug 23;14:219. doi: 10.1186/s13287-023-03398-1 (PMC10464375; doi:10.1186/s13287-023-03398-1)
Supplement: Supplementary file 2 — Additional file 2: Figure S1. Hormone levels evaluation and in vitro cytotoxicity test results. Figure S2. RT-qPCR or/and western blot analysis of DHT-producing enzymes SRD5A2 and DHT-degrading enzymesin hDPCs co-cultured with CEFFE 72 h. Figure S3. Original data of AR western blot results in hair follicles of C57BL/6 mice. Figure S4. Original data of AR western blot results in hDPCs. Figure S5. Original data of SRD5A2 western blot results in hDPCs . [file 13287_2023_3398_MOESM2_ESM.docx]

# *Title:* Cell-free fat extract restores hair loss: a novel therapeutic strategy for androgenetic alopecia

***Authors:*** Yizuo Cai ^1, †^, Zhuoxuan Jia ^1, †^, Yichen Zhang ^2^, Bijun Kang ^1^, Chingyu Chen ^1^, Wei Liu ^1, *^, Wei Li ^1, *^, and Wenjie Zhang ^1, *^

***Institutions:*** ^1^ Department of Plastic and Reconstructive Surgery, Shanghai 9th People's Hospital, Shanghai Jiao Tong University School of Medicine, Shanghai Key Laboratory of Tissue Engineering, National Tissue Engineering Center of China, 639 ZhiZaoJu Road, Shanghai 200011, China; ^2^ Department of Biological and Environmental Engineering, Cornell University

***Co-Author:*** Yizuo Cai; Zhuoxuan Jia

***Corresponding Author****:* ^*^Wei Liu (Email: liuwei_md@126.com); ^*^Wei Li (Email: [liweiboshi@163.com](mailto:liweiboshi@163.com)); ^*^Wenjie Zhang (Email: [wenjieboshi@aliyun.com](mailto:wenjieboshi@aliyun.com))

* Correspondence: [liweiboshi@163.com](mailto:liweiboshi@163.com) (Wei Li); [wenjieboshi@aliyun.com](mailto:wenjieboshi@aliyun.com) (Wenjie Zhang)


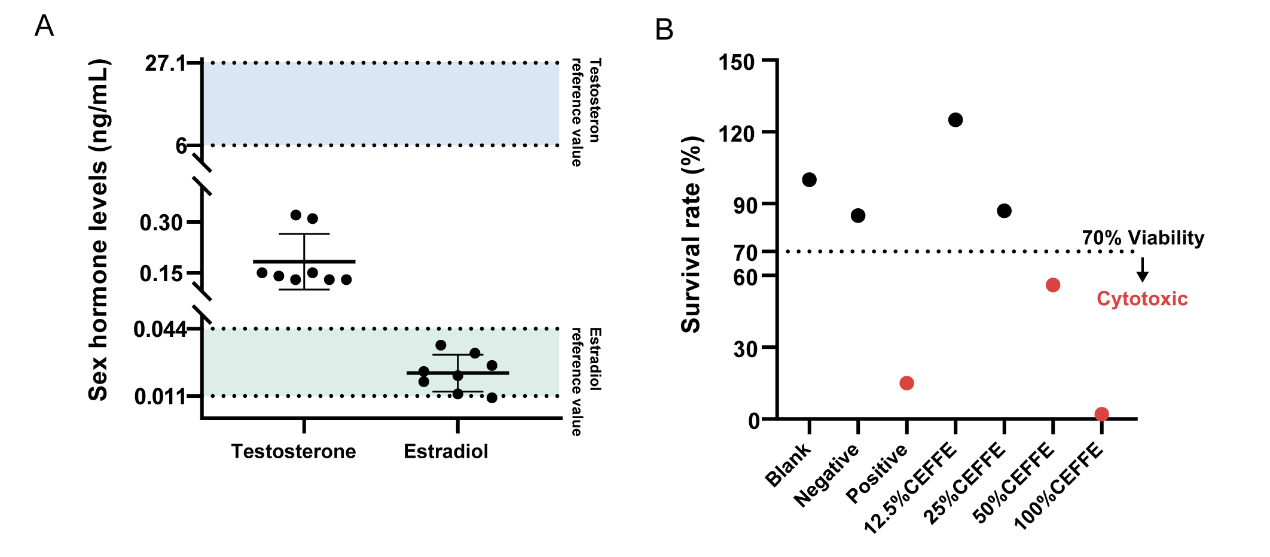


**Supplemental Fig. 1A** Hormone levels evaluation. The blue dotted box indicated the normal range of testosterone (6.0-27.1 ng/mL). The green dotted box indicated the normal range of estradiol (0.011-0.044 ng/mL). **B** In vitro cytotoxicity test. Cell viability less than 70% is considered potentially cytotoxic.


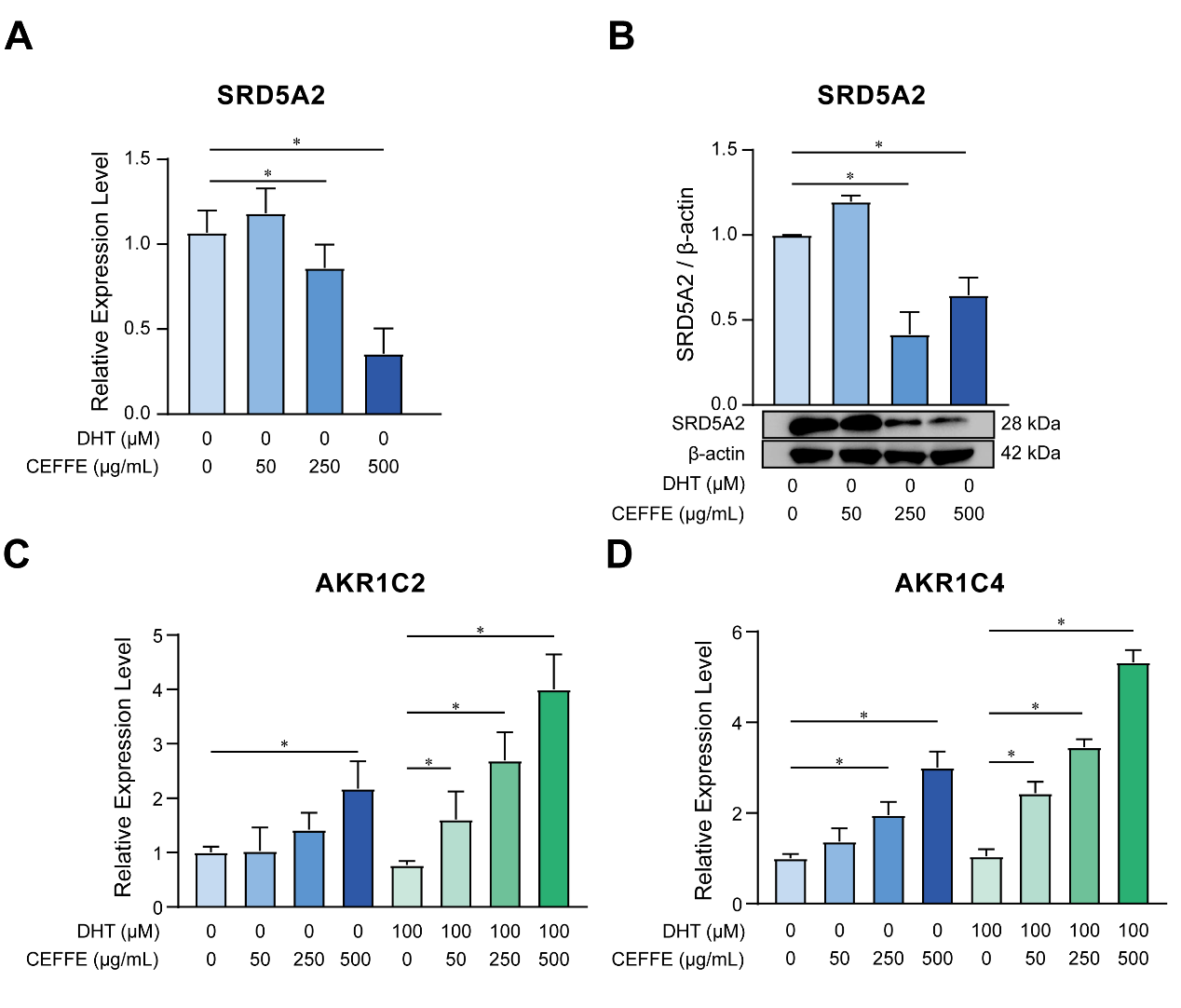


**Supplemental Fig. 2A, B** RT-qPCR and western blot analysis of DHT-producing enzymes SRD5A2 in hDPCs co-cultured with CEFFE 72h. Original data was presented in supplemental Fig. 5. **B** qRT-PCR analysis of DHT-degrading enzymes (AKR1C2 and AKR1C3) in hDPCs co-cultured with CEFFE, DHT and CEFFE+DHT 72h. Data represent the mean ± SD; n = 3; * = p< 0.05.


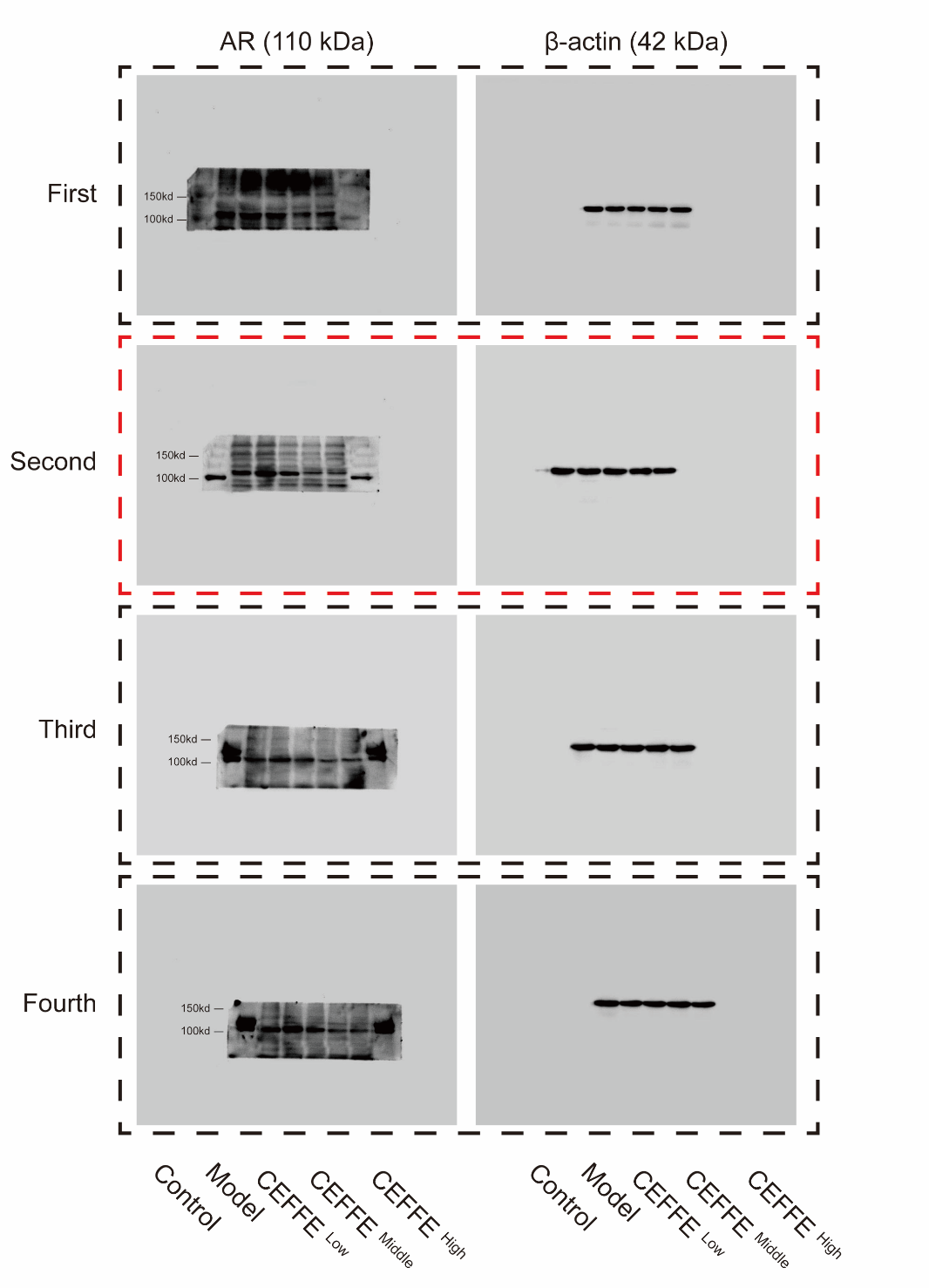


**Supplemental Fig. 3** Original data of AR western blot results in hair follicles of C57BL/6 mice. (Fig. 2G). Images from the same gel was put in the black dashed box. Images presented in the manuscript was put in the red dashed box.


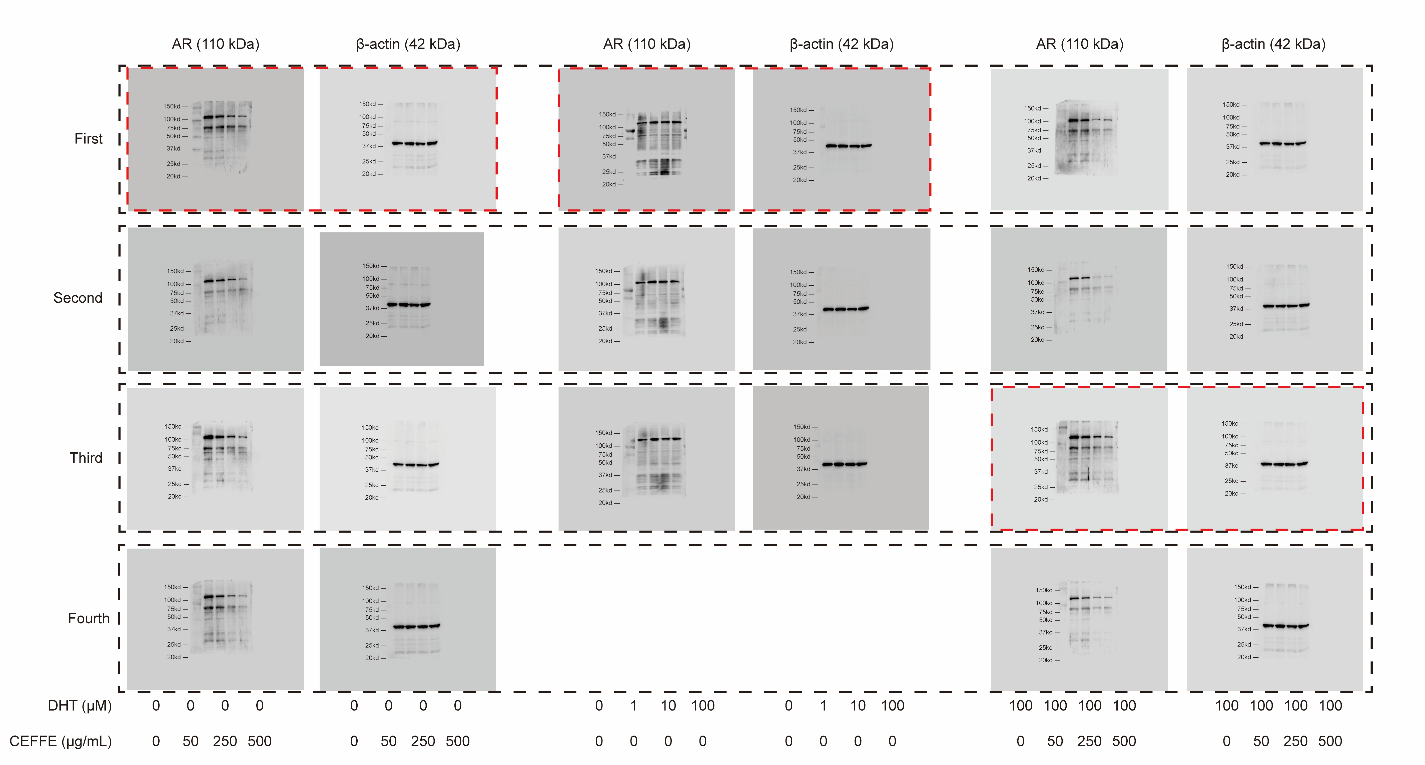


**Supplemental Fig. 4** Original data of AR western blot results in hDPCs (Fig. 5C). Images from the same gel was put in the black dashed box. Images presented in the manuscript was put in the red dashed box.


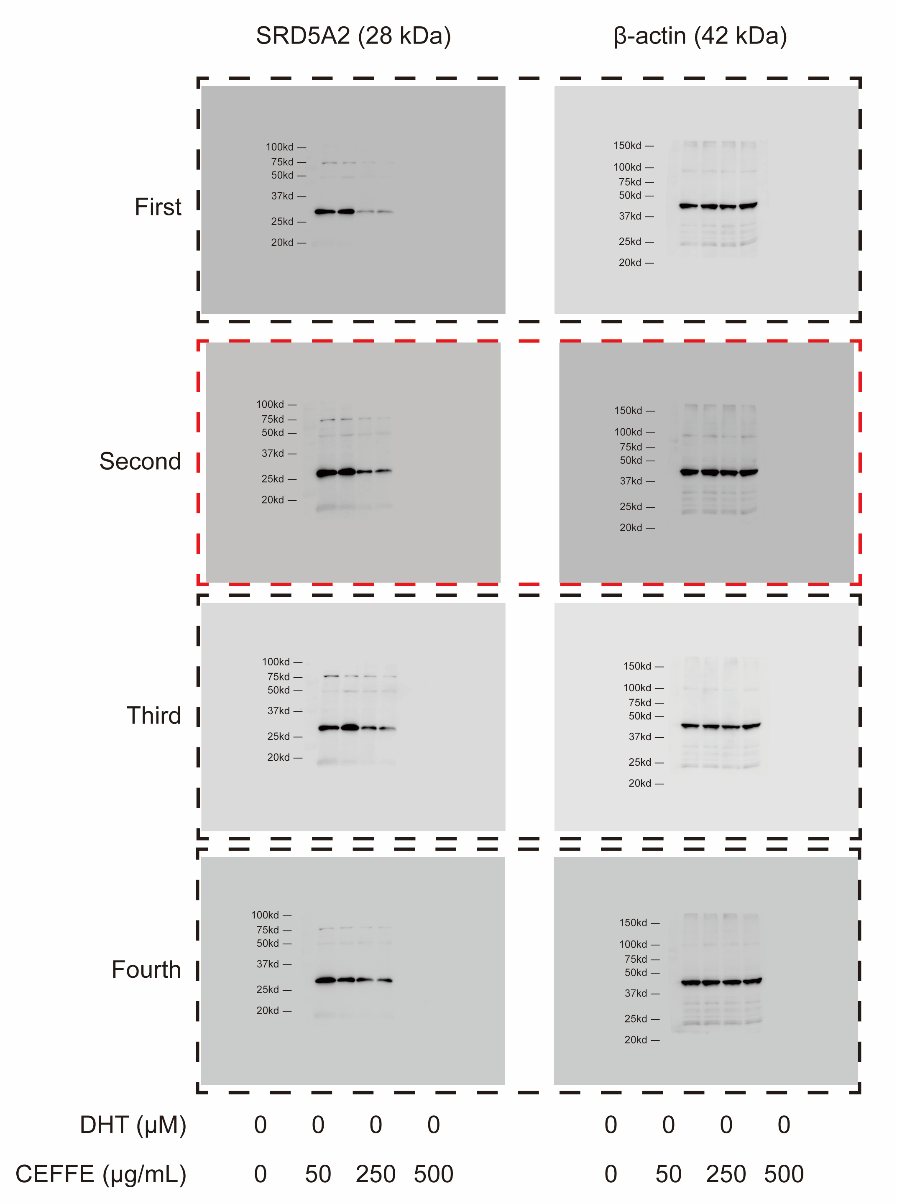


**Supplemental Fig. 5** Original data of SRD5A2 western blot results in hDPCs (Supplemental Fig. 2B). Images from the same gel was put in the black dashed box. Images presented in the manuscript was put in the red dashed box.
